# Identification of circulating tumour cells in early stage breast cancer patients using multi marker immunobead RT-PCR

**DOI:** 10.1186/1756-8722-2-24

**Published:** 2009-06-05

**Authors:** Michael P Raynor, Sally-Anne Stephenson, Kenneth B Pittman, David CA Walsh, Michael A Henderson, Alexander Dobrovic

**Affiliations:** 1 Department of Haematology/Oncology, The Queen Elizabeth Hospital, Adelaide, South Australia 5011, Australia; 2 Department of Medicine, University of Adelaide, The Queen Elizabeth Hospital, Adelaide, South Australia 5011, Australia; 3 Institute of Health and Biomedical Innovation, Queensland University of Technology, Kelvin Grove, Queensland 4059, Australia; 4 Department of Surgery, University of Adelaide, The Queen Elizabeth Hospital, Adelaide, South Australia 5011, Australia; 5 Department of Surgery, University of Melbourne and Peter MacCallum Cancer Centre, Locked Bag 1, A'Beckett St, Melbourne, Victoria, Australia; 6 Department of Pathology, Peter MacCallum Cancer Centre, Locked Bag 1, A'Beckett St, Melbourne, Victoria 8006, Australia; 7 Department of Pathology, University of Melbourne, Parkville, Victoria 3010, Australia

## Abstract

**Introduction:**

The ability to screen blood of early stage operable breast cancer patients for circulating tumour cells is of potential importance for identifying patients at risk of developing distant relapse. We present the results of a study of the efficacy of the immunobead RT-PCR method in identifying patients with circulating tumour cells.

**Results:**

Immunomagnetic enrichment of circulating tumour cells followed by RT-PCR (immunobead RT-PCR) with a panel of five epithelial specific markers (*ELF3*, *EPHB4*, *EGFR*, *MGB1 *and *TACSTD1*) was used to screen for circulating tumour cells in the peripheral blood of 56 breast cancer patients.

Twenty patients were positive for two or more RT-PCR markers, including seven patients who were node negative by conventional techniques. Significant increases in the frequency of marker positivity was seen in lymph node positive patients, in patients with high grade tumours and in patients with lymphovascular invasion. A strong trend towards improved disease free survival was seen for marker negative patients although it did not reach significance (p = 0.08).

**Conclusion:**

Multi-marker immunobead RT-PCR analysis of peripheral blood is a robust assay that is capable of detecting circulating tumour cells in early stage breast cancer patients.

## Introduction

RT-PCR of peripheral blood mononuclear cells (PBMNCs) using lineage-specific markers is the most common published methodology for the detection of circulating tumour cells (CTCs) in the peripheral blood. RT-PCR was first used to detect circulating melanoma [1] and neuroblastoma cells [2]. Due to the high levels of sensitivity necessary to detect rare cancer cells, nested RT-PCR is often used and therefore even low levels of illegitimate transcription in PBMNCs can cause false positive results [3-5]. Nested RT-PCR is also time consuming and stringent procedures need to be observed in order to minimize the risk of false positives due to PCR product cross contamination.

We developed the immunobead PCR methodology using immunomagnetic beads coated with an epithelial cell specific antibody to enrich carcinoma cells from whole blood [6]. When blood from a patient is incubated with antibody-coated beads, the beads attach to any epithelial cells that might be in the blood. The justifiable assumption is that the only epithelial cells in blood or bone marrow are carcinoma cells. A magnet can then be used to harvest these cells. A modification combining immunobead enrichment with RT-PCR detection of lineage-specific markers (immunobead RT-PCR, IB RT-PCR) was subsequently developed [7]. This minimised the problem of illegitimate transcription, allowed the use of whole blood rather than the mononuclear cell fraction, and eliminated the requirement for nested RT-PCR.

We subsequently reported a strategy to identify sensitive and specific RT-PCR assays to be used in immunobead RT-PCR analysis [8]. This method allowed the selection of a panel of RT-PCR markers suitable for immunobead RT-PCR. These included 2 novel markers *ELF3 *(also known as *ESX*) and *EPHB4*, as well as the previously used markers epidermal growth factor receptor (*EGFR*), *TACSTD1 *(also known as epithelial cell adhesion molecule – *EpCAM*) and mammaglobin 1 (*MGB1*). These markers were both sensitive enough to enable detection of a single tumour cell and specific enough not to be amplified from PBMNCs that may contaminate the immunobead-tumour cell pellet. In this new report, we assessed this panel of markers in a prospective study using peripheral blood samples from 56 predominantly early stage breast cancer patients.

## Methods

### Patient samples

Peripheral blood (10 ml) was collected in potassium EDTA tubes from 56 breast cancer patients ranging in age from 37–89 years who presented for pre-admission counselling prior to surgery at The Queen Elizabeth Hospital, Adelaide, Australia. The first 2 ml of blood was discarded to avoid contamination from the skin puncture. Peripheral blood was also collected from 10 normal individuals for use as negative control samples and in reconstruction experiments. Informed consent was obtained in all cases and ethics approval for this study was obtained from The Queen Elizabeth Hospital Ethics of Human Research Committee. The distribution of tumours according to the TNM classification system was 8 *in situ*, 17 Stage I, 21 Stage IIA, 9 Stage IIB, 1 Stage IIIA.

### Cell lines

The breast cancer cell lines MDA-MB-231, MDA-MB-468, MDA-MB-453 and MCF7 were maintained in Dulbecco's Modified Eagle Medium (Invitrogen, Carlsbad, CA) in 75 cm^2 ^tissue culture flasks at 37°C in a 5% CO_2 _environment. The medium was supplemented with 100 U/ml penicillin, 100 μg/ml streptomycin, 160 μg/ml L-glutamine and 10% heat-inactivated foetal bovine serum (CSL, Melbourne, Australia). Cells were collected at < 90% confluency by trypsin digestion and centrifugation for 5 min at 1000 rpm, resuspended in phosphate buffered saline (PBS) and counted using a haemocytometer.

### Reconstruction experiments

MDA-MB-453 cells were diluted in PBS and counted to give aliquots containing 10, 100, and 1000 cells. Triplicate aliquots were seeded into 10 ml of normal blood and analysed by immunobead enrichment and RT-PCR. Normal donor blood with no cells added was used as a negative control.

### Immunobead-enrichment and RT-PCR detection of circulating epithelial cells

The immmunobead RT-PCR technique has been described previously [7]. Briefly, each 10 ml patient blood sample was incubated with 4 million immunomagnetic Dynabeads M-450 (Dynal, Oslo, Norway), labelled with the monoclonal antibody BerEP4 (Dako, Gestrop, Denmark). Each tube was placed on a low speed-rotating mixer for 2 h at 4°C. Bead rosetted cells were isolated in each tube using a magnetic array (Dynal), enabling the beads to be washed 3 times in PBS to remove unbound PBMNCs. The bead/cell isolates were then transferred to a microcentrifuge tube.

The captured cells were then lysed in a 9.5 μl volume of solution containing 0.3% v/v Nonidet P-40 detergent (Sigma), 500 ng random hexamers (Pharmacia, Uppsala, Sweden), 20 U of RNasin (Promega, Madison, WI) and 10 mM DTT, then stored at -80°C until needed for reverse transcription. Reverse transcription was initiated by the addition of 5× First Strand Buffer, 200 U of Superscript II (Invitrogen), 0.5 mM of each deoxynucleotide triphosphate (Roche Applied Science, Mannheim, Germany), with ultra-pure water (Fisher Biotech, Perth, Australia) to a final volume of 20 μl. The reaction was incubated at 42°C for 50 min, and then the reverse transcriptase reaction was inactivated by incubation at 70°C for 10 min.

After reverse transcription, 3.9 μl of cDNA was used as the template in a single round of PCR amplification with 200 nM of each gene specific primer pair (Table 1), 1 U of HotStarTaq (Qiagen, Hilden, Germany), 2.5 mM MgCl_2_, and 200 μM of each deoxynucleotide triphosphate, in the supplied PCR buffer. Cycling conditions included an initial denaturation step at 95°C for 15 min, then 1 min at each of 94°C, 66–68°C and 72°C for 45 – 55 cycles and a final extension of 7 min at 72°C. Amplification products were visualised by ethidium bromide staining following separation by electrophoresis through agarose gels. In each case, a negative control for the RT reaction, made up of the components of the RT reaction mixture with or without lysis mix, and without the addition of RNA, was used as the template in the PCR reaction (no cDNA made and therefore no amplification expected). The PCR negative control contained the reagents of the PCR reaction but lacked template. Cell line cDNA was included as a positive control for the PCR reaction. Genomic DNA (100 ng) was used to confirm that a product of equal size to the cDNA product would not be amplified from the DNA in the cell lysate.

**Table 1:** RT-PCR primers and PCR conditions.

Primer name	GenBank Accession Number	Sequence 5' – 3'	PCR annealing temperature	Size
*ELF3 *s	AF016295	CTCGGAGCTCCCACTCCTCAGA		

*ELF3 *as		GCTCTTCTTGCCCTCGAGACAGT	68°C	188 bp

*EPHB4 *s	AB209644	CCCCAGGGAAGAAGGAGAGCTG		

*EPHB4 *as		GCCCACGAGCTGGATGACTGTG	68°C	250 bp

*EGFR *s	AB209442	TGTGAGGTGGTCCTTGGGAATTTGG		

*EGFR *as		TGCTGACTATGTCCCGCCACTGGA	66°C	339 bp

*TACSTD1 *s	BC014785	GGACCTGACAGTAAATGGGGAAC		

*TACSTD1 *as		CTCTTCTTTCTGGAAATAACCAGCAC	68°C	186 bp

*MGB1 *s	AY217100	CGGATGAAACTCTGAGCAATGTTGAG		

*MGB1 *as		CTGCAGTTCTGTGAGCCAAAGGTC	68°C	110 bp

Sequences are shown for sense (s) and antisense (as) primers. The annealing temperatures used for the PCR reactions and the PCR product sizes are also shown.

### Statistical Analysis

Clinical follow-up was obtained through the Cancer Registry database at The Queen Elizabeth Hospital for the patients enrolled in the study. The database included disease stage (TNM staging system), tumour size and grade, ER/PR status, presence or absence of lymphovascular invasion, and date and cause of death. Frequency data was analysed with the Fisher Exact Test. Metastasis free survival was estimated with Kaplan Meier curves [9] which were compared with the log rank Test [10]. All statistical tests were two sided and p < 0.05 was considered to be statistically significant. All statistical tests were performed using SPSS (version 16 Chicago, Illinois, USA).

## Results

### Sensitivity experiment

**Figure 1:**
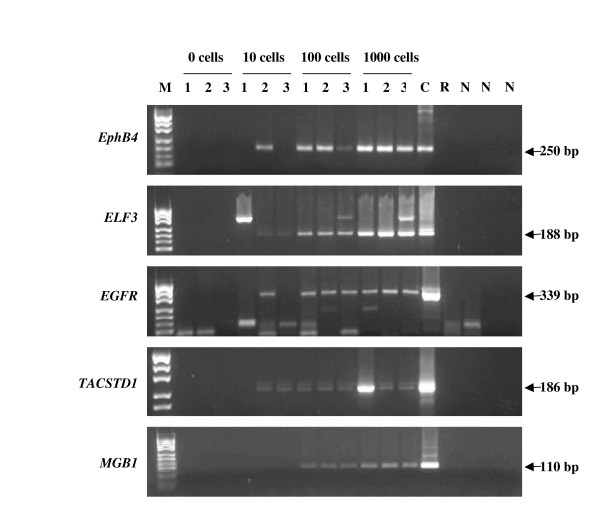
**IB RT-PCR sensitivity test on dilutions of MDA-MB-453 in normal blood**. Cells were added to 10 mls of normal blood. Lane 1, M = *pUC*19/*Hpa*II marker. Lanes 2–4, no added cells. Lanes 5–7, 10 added cells. Lanes 8–10, 100 added cells, Lanes 11–13, 1000 added cells. Lane 14, C = cDNA positive control. Lane 15, R = RT negative control. Lanes 16–18, N = PCR negative controls. Product size is shown in base pairs (bp). A genomic DNA band is seen above the RT-PCR band for *ELF3*. The legend shows cells/10 ml of blood.

Ten, hundred and thousand cell aliquots of the MDA-MB-453 cell line were seeded into triplicate ten ml tubes containing blood from a single normal donor and evaluated for the sensitivity of detection of the five immunobead RT-PCR markers (Figure 1). Blood samples containing no added cells were negative for all markers. Blood samples containing an estimated 100 or 1000 seeded cells were positive for all 5 markers in 3/3 replicates. In samples containing an estimated 10 seeded cells, *TACSTD1 *was detected in 2/3 replicates, *ELF3 *was detected in 2/3 replicates, *EGFR *was detected in 1/3 replicates, *EphB4 *was detected in 1/3 replicates while *MGB1 *was not detected. No other markers were positive in the samples that were negative for *TACSTD1 *suggesting that no MDA-MB-453 cells were captured with the BerEp4-conjugated beads from these samples as *TACSTD1 *codes for EpCAM which BerEp4 recognises.

### Immunobead RT-PCR analysis of blood samples

The expression of each of the five RT-PCR markers was assessed in the immunomagnetically enriched fraction of blood samples obtained prior to surgery from 56 early stage breast cancer patients (Table 2). One hundred nanograms of cDNA from MDA-MB-468, MCF7, MDA-MB-453, or MDA-MB-231 breast cancer cell lines were used as positive controls for each RT-PCR assay.

**Table 2:** Clinical details and RT-PCR positivity data.

	**Stage**	**T**	**N**	**ER**	**PR**	**Grade**	**LVI**	***TACSTD1***	***EPHB4***	***ELF3***	***EGFR***	***MGB1***
1	*in situ*	*Tis*	-	x	x	ND	-	-	-	-	-	-

2	*in situ*	*Tis*	-	+	+	ND	-	-	-	-	-	-

3	*in situ*	*Tis*	-	-	-	ND	-	-	-	-	-	-

4	*in situ*	*Tis*	-	+	+	ND	-	-	-	-	-	-

5	*in situ*	*Tis*	-	x	x	ND	-	-	-	-	-	-

6	*in situ*	*Tis*	-	x	x	ND	-	-	-	+	-	-

7	*in situ*	Tis	-	x	x	ND	-	+	+	+	+	-

8	*In situ*	*Tis*	x	+	+	2	-	-	-	-	-	-

9	I	T1	-	+	+	1	-	-	-	-	-	-

10	I	T1	-	+	-	1	-	-	-	-	-	-

11	I	T1	-	+	+	1	-	-	-	-	-	-

12	I	T1	-	+	+	1	-	-	-	-	-	-

13	I	T1	-	+	+	1	-	-	-	-	-	-

14	I	T1	-	+	+	1	-	-	-	-	-	-

15	I	T1	-	+	+	1		-	-	-	-	-

16	I	T1	-	+	+	1	-	+	-	-	-	-

17	I	T1	-	+	+	1	-	+	+	-	+	-

18	I	T1	-	+	+	2	-	-	-	-	-	-

19	I	T1	-	+	+	2	-	-	-	-	-	-

20	I	T1	-	-	+	2	-	-	-	-	-	-

21	I	T1	-	+	+	2	-	-	-	-	-	-

22	I	T1	-	-	-	3	-	+	+	-	-	-

23	I	T1	-	+	+	3	-	-	-	-	-	-

24	I	T1	-	+	+	3	+	+	+	+	-	-

25	I	T1	-	-	+	ND	-	-	-	-	-	-

26	IIa	T1	+	+	-	1	-	+	+	+	+	+

27	IIa	T1	+	+	+	1	-	-	-	-	-	-

28	IIa	T1	+	+	+	2	-	+	+	+	+	+

29	IIa	T1	+	+	+	2	-	+	+	+	+	+

30	IIa	T1	+	+	+	2	+	+	+	+	+	+

31	IIa	T1	+	x	x	2	+	-	-	-	-	-

32	IIa	T1	+	+	+	2	-	-	-	-	-	-

33	IIa	T1	+	-	+	3	-	+	+	+	+	-

34	IIa	T2	-	x	x	1	-	-	-	-	-	-

35	IIa	T2	-	+	+	1	-	-	-	-	-	-

36	IIa	T2	-	+	+	1	-	-	-	-	-	-

37	IIa	T2	-	+	+	1	-	-	-	-	-	-

38	IIa	T2	-	+	+	1	+	+	+	+	-	-

39	IIa	T2	-	+	+	1	+	+	+	+	+	-

40	IIa	T2	-	+	+	2	-	-	-	-	-	-

41	IIa	T2	-	+	+	2	-	-	-	-	-	-

42	IIa	T2	-	+	+	2	+	-	-	-	-	-

43	IIa	T2	-	+	+	2	-	-	-	-	-	-

44	IIa	T2	-	+	+	2	+	-	-	-	-	-

45	IIa	T2	-	-	-	3	-	-	-	-	-	-

46	IIa	T1	-	+	+	2	-	-	-	-	-	-

47	IIb	T2	+	+	+	1	-	+	+	+	+	-

48	IIb	T2	+	x	x	2	+	+	+	+	+	+

49	IIb	T2	+	+	+	3	+	+	+	+	+	+

50	IIb	T2	+	+	+	3	-	+	+	+	+	+

51	IIb	T2	+	+	-	3	+	+	+	+	+	-

52	IIb	T2	+	-	-	3	+	-	-	-	-	-

53	IIb	T3	-	-	-	3	+	+	+	+	+	-

54	IIb	T1	x	-	-	2	+	+	+	+	+	+

55	IIb	T2	x	-	-	3	+	+	+	+	+	-

56	IIIa	T3	+	x	x	2	-	+	+	+	+	-

Stage, tumour size (T), nodal status (N), estrogen receptor status (ER) and progesterone receptor status (PR), lymphovascular invasion (LVI), tumour grade, and positive or negative expression of the 5 RT-PCR markers are shown for the 56 breast cancer patients. ND denotes no data. x denotes unknown.

In 20 cases, at least two markers were positive (36%). Of these 20 cases, 8/20 (40%) showed expression of all five markers, 8 (40%) were positive for four markers, 3 (15%) were positive for three markers, and 1 case (5%) was positive for two markers. Two patients, one positive for *ELF3 *only (patient 6), and one for *TACSTD1 *only (patient 15) were excluded from analysis because it was considered unlikely these results were due to disseminated disease (see Discussion). Blood samples from 10 normal donors were analysed in an identical manner to those blood samples obtained from breast cancer patients, and were found negative for all RT-PCR markers (data not shown).

### Expression of RT-PCR markers by stage

The expression of RT-PCR markers was evaluated by clinical stage for the 56 patient samples (Table 2, column designated Stage). Positive RT-PCR marker expression was detected in 1/8 (12.5%) patients considered to have *in situ *disease, 3/17 (17.6%), patients with Stage I disease, 7/21 (33.3%) patients with Stage IIa disease, 8/9 (88.8%) patients with Stage IIb disease and the one patient with Stage IIIa disease.

In a comparison of *in situ *and stage I patients *versus *Stage IIa and Stage IIb patients, there was a statistically significant increase in the frequency of marker positivity in the higher stage patients (p = 0.008). There was also a statistically significant increase in the frequency of marker positivity for Stage IIa *versus *Stage IIb patients (p = 0.007).

### Expression of RT-PCR markers by tumour size

Nine of the 28 patients (32%) that had tumours ≤ 2 cm in greatest dimension (T1) were RT-PCR positive (Table 2, column T). Eight of the 19 patients (42%) that had tumours > 2 cm but not more than 5 cm (T2) were RT-PCR positive. Both patients with tumours > 5 cm (T3) were RT-PCR positive. When *in situ *and T1 patients were compared to T2 and T3 patients, the observed trend to increasing numbers of patients that were positive for PCR markers did not reach significance (p = 0.08).

### Expression of RT-PCR markers by lymph node status

Lymph node involvement for all patients in the study had been assessed using haemotoxylin and eosin staining (Table 2). Certain patients had their sentinel lymph nodes also assessed by immunohistochemistry. In total, 15/56 (27%) patients showed metastasis to one or more lymph nodes. Eleven of the 15 (73%) patients with positive lymph nodes were also positive for expression of RT-PCR markers in blood. Two of three patient's where lymph node involvement could not be assessed, were marker positive. Importantly, 7/41 (17%) patients who were considered node negative by conventional techniques were positive for at least two of the RT-PCR markers, with six of these patients showing positive expression of three or more markers. Nevertheless, lymph node positive patients were more likely to be RT-PCR positive (p = 0.00015).

### Expression of RT-PCR markers by grade

Of the 56 patients, 18 were classified as having Bloom and Richardson Grade I (well differentiated) tumours, 20 were classified as Grade II (moderately differentiated) tumours and 12 were classified as Grade III (poorly differentiated) tumours (Table 2). For six patients, Bloom and Richardson grading was not available. Five of 18 (28%) Grade I patients, 6/20 (30%) Grade II patients, 8/12 (66%) Grade III patients, and 1/6 patients where tumour grading was not available, were RT-PCR positive. RT-PCR marker positivity was statistically higher (p = 0.01) in patients with Grade 3 tumours compared to those with Grade 1 and 2 tumours.

### Expression of RT-PCR markers by lymphovascular invasion (LVI)

Fourteen patients were determined to have LVI present (Table 2, column LVI) and 10/14 (71%) were RT-PCR positive. LVI was not observed in 42 patients and of these, 10/42 (24%) were RT-PCR positive. The association of LVI and RT-PCR positivity was statistically significant (p = 0.002). LVI did not appear to be associated with grade, nodal status, or ER/PR status. There was however, a tendency for LVI to be associated with stage of disease and tumour size with the majority of patients that were LVI positive being Stage IIa or above with tumours classified as T2.

### Expression of RT-PCR markers by ER/PR status

ER/PR status was available for 48 of the 56 patients. Of the 38 ER positive patients, 12 (31%) were positive for RT-PCR markers and 5/10 (50%) of the ER negative patients were positive. Of the 38 PR positive patients, 11 (29%) were marker positive, and of the 10 PR negative patients, 6 (60%) were marker positive. There was no significant association with ER status and marker positivity (p = 0.2) although there was a trend towards marker positivity in ER negative patients. There was a marginally significant association found between marker positivity and PR status (p = 0.04).

### Individual RT-PCR Marker expression

**Figure 2:**
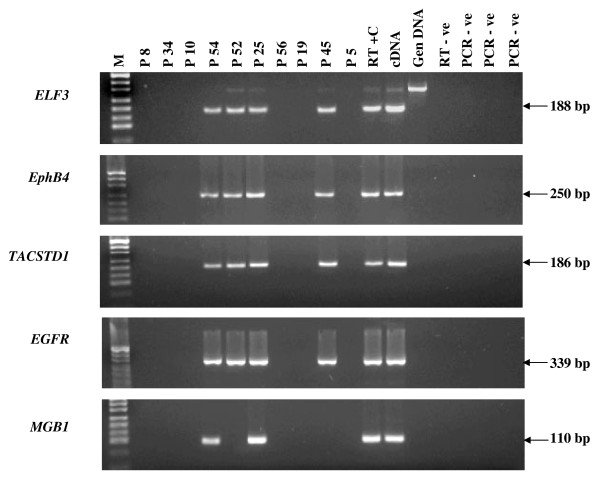
**IB RT-PCR analysis for a representative group of 10 breast cancer patients**. Patient number corresponds to numbers provided in Table 2. Product sizes are shown in base pairs. M = *pUC*19/*Hpa*II marker. A genomic DNA band is seen above RT-PCR band for *ELF3*.

Transcripts corresponding to *TACSTD1 *and *EPHB4 *were amplified in 20/20 (100%) of cases. Transcripts corresponding to *ELF3 *were amplified from 18/20 (90%), *EGFR *from 17/20 (85%), and *MGB1 *transcripts were amplified from 8/20 (40%) of samples. There appeared to be no strong association with the individual expression of *TACSTD1*, *EPHB4*, *ELF3 *and *EGFR *and any of the prognostic factors examined above (data not shown). A representative group of patients positive for IB RT-PCR marker expression is presented in Figure 2.

### Survival analysis

**Figure 3:**
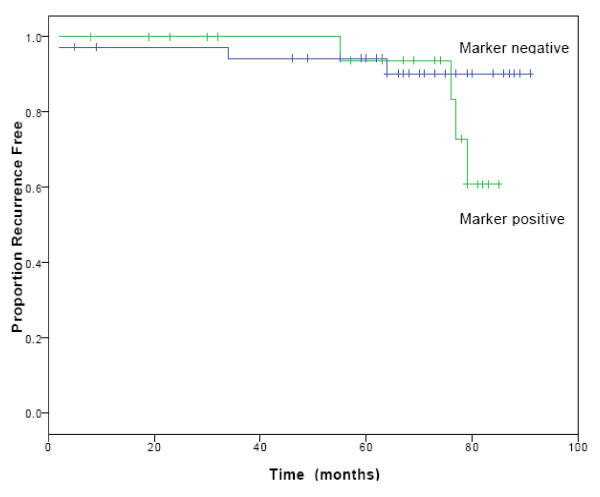
**Disease free survival for 56 patients with early stage breast cancer**. Comparing patients positive for RT-PCR markers (2 or more) with patients negative for RT-PCR markers (0 or 1).

Kaplan-Meier survival analysis was performed using recurrence or death from disease as endpoints. The log-rank test was used to compare survival curves of breast cancer patients positive or negative for RT-PCR markers. There was no significant difference between the 2 groups (p = 0.09) (Figure 3) but a strong trend was seen towards poorer survival for patients positive for RT-PCR markers.

## Discussion

Identification of epithelial cells in the peripheral blood of patients with breast cancer may be used to identify patients in whom haematogenous dissemination of tumour cells has occurred. However, the prognostic relevance of CTCs in the blood of patients with early stage disease, without overt metastasis, is still under investigation. Several studies have suggested that with the development of improved detection techniques, detection of CTCs will provide significant prognostic information [reviewed in [11-15]].

Many recent studies have aimed to improve the methods of detection and finding new markers for CTCs. Multi-marker RT-PCR assays have become widely used for detecting both lymph node involvement and CTCs in breast cancer patients [e.g. [15-22]]. We previously reported a panel of markers that allowed the sensitive and specific identification of a single breast cancer line cell, even if isolated with as many as 100 contaminating haematopoietic cells [8]. We have now estimated the sensitivity of detection in reconstruction experiments using immunobead capture of the MDA-MB453 breast cancer cell line diluted into blood samples followed by RT-PCR for the panel of markers. Seeding dilutions of MDA-MB453 into normal blood resulted in consistent detection of all 5 RT-PCR markers at a level of 10 cells per ml of blood (Figure 1). In samples containing 1 cell per ml of blood (10 cells total), marker expression was detected in 2/3 samples indicating some loss of cells during the immunobead isolation.

In the main part of this study, the expression of the panel of 5 RT-PCR markers (*TACSTD1*, *EPHB4*, *ELF3*, *EGFR*, and *MGB1*) was determined after immunobead enrichment of circulating epithelial cells in blood samples obtained from 56 early stage breast cancer patients. Circulating epithelial cells were isolated from blood samples based on their ability to bind to the immunobeads *via *the BerEP4 antibody that recognises the TACSTD1 (EpCAM/EGP2) glycoprotein. Therefore, it was determined that patient samples would only be considered positive for circulating cells if *TACSTD1 *expression was positive along with expression of one of the other markers.

Using this panel of markers, 20/56 (36%) of patient blood samples had detectable levels of gene expression for at least two of the markers including *TACSTD1*. As these markers were previously shown to give sensitive and specific identification of tumour cells [8], it can be considered that these patients must have had at least one epithelial cell in that blood sample. Importantly, immunobead isolates from 10 ml blood samples obtained from 10 normal donor individuals with no seeded cells were negative for expression of all markers.

Although the IB RT-PCR method is highly sensitive, it is possible that some cells may not be isolated by the immunobeads due to either the death of the cell during immunobead incubation or lack of expression of the EpCAM target antigen. In addition, it is likely there is some heterogeneity in marker gene expression (where some cells may not be expressing a particular gene at that time) in the captured cells. This is demonstrated in the results of this study where not all of the samples that are positive for at least two markers are positive for all five markers and this highlights the need for the use of multiple markers.

*TACSTD1 *and *EPHB4 *were expressed in 100% of samples that were positive for two or more markers. As *TACSTD1 *encodes EpCAM, the target antigen for the BerEP4 antibody, it was expected that all captured tumour cells would express this gene. Previous studies evaluating *TACSTD1 *expression as a marker of micrometastasis in breast cancer reported expression of this gene in bone marrow and peripheral blood cells of normal individuals [23-25]. However, those studies used nested-RT-PCR without prior immunobead enrichment. In this study, *TACSTD1 *was an excellent control marker for use with IB RT-PCR with none of 10 normal control samples expressing the gene after immunobead "enrichment".

*EPHB4 *has not been used as a marker of disseminated breast tumour cells prior to this study. It has been used in a previous immunobead RT-PCR study screening peripheral blood and lavage samples from patients with colorectal cancer [26]. Wu et al (2004) used immunohistochemistry of 94 tumour tissues to show that 82% of breast tumours had moderate to strong expression of EPHB4 and that this was increased with clinical stage and histological grade [27]. More recently, siRNA and antisense studies have confirmed that EPHB4 has an essential role in many processes that contribute to cancer cell survival and spread in several cancers including breast cancer [28]. *EPHB4 *was found to be an exceptional marker in the present study, with 100% of positive patient samples showing expression of the gene

*EGFR *and *ELF3 *(*ESX*) were expressed in the majority of positive patient samples (81% and 90% respectively). Both these genes have been reported to be over-expressed in breast cancer [29-31]. *EGFR *has been widely used for RT-PCR detection of CTCs [32-38]. The majority of these studies found *EGFR *to be highly specific for CTCs with no expression detected in normal control samples. EGFR seems to be particularly associated with basal type breast cancers and also may be a marker of the epithelial to mesenchymal transition often found in CTCs.

The final marker used in this study was *MGB1 *which has been frequently used for detecting CTCs in breast cancer patients due to its exclusive expression in breast tissue [15,21,39-44]. In most of these reports, detection of *MGB1 *was highly sensitive and specific with detection ranging from 11% to 60% of patient samples. In this study, *MGB1 *performed poorly as a marker of dissemination in comparison to the other 4 markers. Why *MGB1 *expression was not as readily detected as other markers is unclear, but *MGB1 *was also the least frequently expressed marker in the single cell assay reported previously [8]. It is possible that as *MGB1 *is a marker of mammary differentiation it may not be as highly expressed in breast tumours with a relatively undifferentiated phenotype as the other markers used here. This hypothesis is supported by a study that found a significant association (p = 0.020) between absence of *MGB1 *mRNA and grade 3 breast cancers [44]. Interestingly, *MGB1 *expression was not seen in the seven RT-PCR marker positive patients with node negative disease and suggests there is either a low tumour cell burden in the circulation of these patients or there are differences in the biology of node negative tumours.

The relationships between positive RT-PCR marker expression and prognostic indicators were analysed using Fisher's exact test. As the TNM classification system determines stage of disease using tumour size, involvement of lymph nodes, and distant metastasis, the relationship of overall stage of disease and positive expression of markers was examined. Positive results were seen in all stages of disease and included a patient considered to have *in situ *disease. Analysis showed there were significant associations with marker positivity with more advanced stage of disease (*in situ *and Stage I *versus *Stage II p = 0.02) and even within a stage (Stage IIa *versus *Stage IIb p = 0.03).

Tumour size was not strongly associated with marker positivity suggesting that even small tumours can shed cells into the circulation. Similar results regarding tumour size have also been observed in studies of breast cancer patients with tumour cells in their bone marrow [45,46]. In contrast, a study using a quantitative RT-PCR multi-marker assay of blood with tumour specific markers reported a strong correlation with both clinical stage of disease and tumour size [47].

Patients with Grade 3 tumours were more likely to be marker positive in this study than patients with more differentiated tumours. The majority (40%) of RT-PCR positive patients had Grade 3 tumours.

Lymph node involvement was strongly associated with marker positivity, Lymph node involvement has also been associated with the presence of micrometastatic cells in the bone marrow [48]. However, a significant proportion (17%) of node negative patients had CTCs detected by IB RT-PCR. This is lower than the proportion (26%) of node negative patients that had bone marrow micrometastases [48]. These rates are similar to the reported 20–30% of node negative patients that subsequently relapse or die.

The data from this study support the concept that LVI is a good predictor that tumour cells are likely to have entered the circulation. However, CTCs can be detected among patients without evidence of lymphatic and or vascular invasion (25% compared to 75% in lymphatic and or vascular invasion positive group). A report of 1,258 patients evaluated the absence or presence of LVI for any significance towards assessing survival [49]. Presence of LVI was found to be associated with a significantly worse survival based on 12 year follow-up for those with lymph node negative disease and an even worse survival for those with positive nodes. It was suggested that both LVI and lymph node status were highly independent and combined together would be significant predictors of outcome.

Disease-free survival was not found to be significantly associated with marker status however a definite trend towards poorer disease free survival was observed. It is likely that longer follow-up will reveal a statistically significant survival disadvantage for these patients.

The purpose of this study was to evaluate the methodology of detection of CTCs in patients with operable breast cancer. The results are compatible with the hypothesis that CTCs are indicative of a higher risk of recurrence and poorer survival even though statistical significance was not reached. A limitation of the current study is that no reference (housekeeping) genes were used when the study was performed. This does not allow us to conclusively eliminate the possibility that there may have been a small proportion of false negative results. Nevertheless, the multi-marker IB RT-PCR assay has been shown to sensitively detect CTCs in early stage breast cancer patients. A larger prospective study of minimal tumour burden in blood, bone marrow and lymph nodes should provide conclusive evidence of the role of detection of blood-borne CTCs in early stage breast cancer.

## Abbreviations

PCR: polymerase chain reaction; RT-PCR: reverse transcriptase polymerase chain reaction; CTCs: circulating tumour cells; PBMNCs: peripheral blood mononuclear cells; IB RT-PCR: immunobead RT-PCR; PBS: phosphate buffered saline; ER: estrogen receptor; PR: progesterone receptor; TNM: tumour node metastasis; LVI: lymphovascular invasion; *TACSTD1*: tumour-associated calcium signal transducer 1 (also known as epithelial cell adhesion molecule, EpCam,); *ELF3*: E74-like factor 3 (ets domain transcription factor, epithelial-specific), (also known as *ESX*, epithelial specific with serine box); *EPHB4*: EPH receptor B4; *EGFR*: epidermal growth factor receptor; *MGB1*: mammaglobin 1; Tm: melting temperature; bp: base pairs; s: sense; as: antisense.

## Competing interests

The authors declare that they have no competing interests.

## Authors' contributions

MR performed the majority of the experiments, analysed the data and drafted the manuscript. SS assisted with the experiments and the analysis of the data and assisted with the manuscript. DCAW and KP assisted with the design of the project, provided access to clinical samples and were involved in interpreting the data and reviewing the manuscript. MH assisted with the statistical interpretation of the data and reviewed the manuscript. AD was responsible for the overall conception and design of the project, for interpretation of the data and co-writing of the manuscript.
